# Trends in adolescent first births in five countries in Latin America and the Caribbean: disaggregated data from demographic and health surveys

**DOI:** 10.1186/s12978-018-0578-4

**Published:** 2018-08-29

**Authors:** Sarah Neal, Chloe Harvey, Venkatraman Chandra-Mouli, Sonja Caffe, Alma Virginia Camacho

**Affiliations:** 1 Department of Social Statistics and Demography, University of Southampton, Southampton, UK; 2 Human Reproduction Programme/Department of Reproductive Health and Research, World Health Organization, Geneva, Switzerland; 3 Pan American Health Organization, 525 23rd Street, NW, Washington DC, USA; 4 SRH Team, Latin America and the Caribbean Regional Office, United Nations Population Fund, Panama City, Panama

**Keywords:** Adolescent, Sexual health, Pregnancy, Latin America and the Caribbean

## Abstract

**Background:**

Adolescents in the Latin American and Caribbean region continue to experience poor reproductive health outcomes, including high rates of first birth before the age of 20 years. Aggregate national level data fails to identify groups where progress is particularly poor. This paper explores how trends in adolescent births have changed over time in five countries (Bolivia, Colombia, Dominican Republic, Haiti, and Peru) using data disaggregated by adolescent age group, wealth and urban / rural residence.

**Methods:**

The study draws on Demographic and Health Survey data from five countries where three surveys are available since 1990, with the most recent after 2006. It examines trends in adolescent births by wealth status and urban/rural residence.

**Results:**

There has been little progress in reducing adolescent first births over the last two decades in these countries. Adolescent first births continue to be more common among the poorest and rural residents, and births among the youngest age-group (< 16 years) are particularly concentrated among these populations.

**Conclusion:**

Adolescent first births continue to be a major issue in these five countries, including amongst the youngest group (< 16 years), although the contexts in which it is occurring are changing over time. Efforts are needed to expand sexual education and services for adolescents and young people, as well as introduce and enforce legislation to provide effective protection from abuse or exploitation. Greater disaggregation of adolescent fertility data is needed if we are to measure progress towards the attainment of the Sustainable Development Goals to “leave no-one behind”.

## Plain English summary

Adolescent girls in Latin America and the Caribbean continue to be at risk of poor reproductive health and have a high risk of giving birth before the age of 20 years. This study analyses survey data from Bolivia, Colombia, Dominican Republic, Haiti, and Peru to examine trends in the percentage of adolescents giving birth before age 20 years.

We find that little progress has been made in reducing adolescent first births in these countries. Births to young women are much more common among the poorest and those living in rural areas, and this is particularly true for young women who give birth at very young ages (e.g. before 16 years).

Our study highlights the need to improve sexual health education and services for adolescents and young people, as well as ensure legal frameworks to protect them.

## Background

Young people in Latin America and the Caribbean (LAC) continue to face significant barriers to their sexual and reproductive health, including adolescent pregnancy. Around 15% of all births in this region are to women below the age of 20 years [1], with an adolescent fertility rate (15–19 years) of 65 per 1000 in 2014 [2]. Teenage pregnancy is associated with a number of adverse health outcomes for both mother and child [3, 4], as well as social and economic disadvantages for individuals and families [5]. The associated loss of productivity and negative impacts on development at the national level [6] has led policy-makers to identify it as an important area for intervention. Sexual behaviour for both male and female adolescents is underpinned by complex socio-economic, educational, cultural, geographical and service-availability factors, and contexts, patterns, and trends may vary markedly for different populations within countries. However, anaysis of data disaggregating populations by socio-economic and other characteristics is rarely carried out, meaning it is difficult to identify those groups who may be making poor progress. Disaggregated data that examines patterns and trends for different groups is valuable in enabling programmes to target those most at risk, and to ensure that the ambition of the Sustainable Development Goals to “leave no-one behind” is achieved. In particular, greater focus on younger adolescents is important, as they face greater health disadvantages for both mother and infant [4, 7, 8] and may require specific approaches and strategies to address pregnancies in this particularly vulnerable group [9–11].

Overall, adolescent pregnancies in LAC are higher than for other regions with similar levels of development, and the relatively poor progress is at odds with the sharp decline in overall fertility. While the age specific fertility rate (ASFR) 15–19 years fell somewhat from 84 per 1000 in 1990 to 65 per 1000 in 2015, total fertility rate (TFR) had fallen sharply in the same period from 3.2 to 2.1 births per woman [12]. In addition, reliance on the adolescent fertility rate does not fully illustrate the issue: while it has fallen somewhat, the percentage of women who enter motherhood before the age of 20 years has actually increased since 1990 [13]. The reduction in adolescent fertility is driven by young mothers having fewer subsequent births, rather than fewer adolescent women becoming mothers at all [13]. It is therefore important to identify and highlight patterns in first births as this illustrates more clearly trends in the proportion of young women exposed to the disadvantages of adolescent motherhood.

Existing literature suggests that the persistence of adolescent fertility reflects a range of cultural and socio-economic factors. While much of the region has benefited from increased economic growth and social development, these benefits are not experienced equally. Even though educational opportunities have increased for young people, this is often not reflected in labour market prospects, particularly for young women [14]. This trend means that many young women (particularly within poorer communities) may continue to have low expectations about their futures in terms of employment, which makes avoiding adolescent pregnancy appear less important [15]. While progress has been made in promoting gender equity within many important aspects of life and in particular education, persistent barriers exist for women in expanding their economic opportunities and exercising agency and choice over their lives [14]. Cultural expectations of early motherhood are also often cited as important drivers, and it is stated that adolescent girls in LAC (particularly amongst the poorest groups and indigenous populations), desire and plan for early motherhood, with early partnering and an assumption of childbearing continuing to be the norm [16]. However, more recent discourses suggest that the fertility desire aspect of adolescent pregnancy has been overplayed, and the region has demonstrated a sharp decline in the proportion of adolescents who report they wanted their pregnancy [17].

This paper addresses how patterns of adolescent^1^ first births in women have changed in five countries within the LAC region: Bolivia, Colombia, Dominican Republic, Haiti and Peru. It addresses two questions:

- What is the age distribution of first births to adolescents, and have the percentages of women entering adolescent motherhood in each age group changed since 1990?
- How do the socio-economic characteristics (wealth and urban / rural residence) of young women having a first adolescent birth differ between younger and older adolescents and how have these characteristics changed over time?

These five countries represent a considerable geographic spread within the region, with two from the Caribbean region and the rest within South America. ASFR 15–19 years based on the most recent Demographic and Health Survey (DHS) range from 64 per 1000 (Peru) to 90 per 1000 (Dominican Republic).

The persistence of high adolescent fertility and concerns about negative repercussions for both families and wider society in these five countries has led to increased concern among governments, non-governmental organisations and the media [18, 19]. Developing effective interventions requires a nuanced and in-depth understanding of trends and patterns in adolescent births, which involves disaggregation of data by sub-groups, as well as understanding the context in which births are occurring and how this may be changing.

## Methods

The analysis is based on Demographic and Health surveys (DHS), which provide nationally representative data comparable across time and place. The five countries were chosen as they had three available surveys carried out since 1990, with the most recent being after 2006. The use of three surveys enabled us to be more confident about the quality of data, as any sharp differences may suggest data inconsistencies. However, rates of change are not presented for each period as originally intended as the change is so small for all countries.

The countries represent markedly different levels of socio-economic development, and Table 1 provides details of their ASFR 15–19 years, TFR, % female literacy and gross national income per capita (GNI). It is interesting to note that while Haiti has by far the lowest GNI and female literacy, it has one of the lowest ASFR 15–19 years (66 per 1000). Conversely, the Dominican Republic has the highest GNI per capita and near-universal female literacy, yet has the highest ASFR 15–19 years. This lack of clear association between wealth at national level and adolescent pregnancy rate has been identified elsewhere [14] and highlights the complex relationship between socio-economic factors and early motherhood within the region. The countries also represent very marked ethnic and cultural diversity. Table 1 also highlights that during the period covered in this study there have been some declines in ASFR 15–19 years in three countries (Bolivia, Dominican Republic and Haiti), a slight increase in Peru, and quite a marked increase in Colombia.

**Table 1:** ASFR 15–19 years, TFR, female literacy and GNI per capita for five study countries

	Age specific fertility rate: 15–19: Baseline (year of survey in brackets)	Age specific fertility rate: 15–19: final survey (year of survey in brackets)	Total fertility rate 15–49^a^	Women who are literate^a^ aged 15–49	GNI per capital PPP (current international $)^b^
Bolivia	94 (1994)	88 (2008)	3.5	92.7	6710
Colombia	70 (1990)	84 (2010)	2.1	–	13,550
Dominican Republic	112 (1996)	90 (2013)	2.5	93	13,600
Haiti	76 (1994)	66 (2012)	3.5	73.6	1760
Peru	61 (1991)	64 (2012)	2.6	94.6	12,060

^a^Data from DHS Statcompiler http://statcompiler.com/en/:For Total fertility it is for most recent survey

^b^Data from The World Bank http://data.worldbank.org/indicator/NY.GNP.PCAP.PP.CD: 2015

The analysis was based on the cohort aged 20–24 years as they will have been exposed to the whole period of interest in the study. In addition, in some countries there is evidence of under-reporting of very early adolescent births among respondents aged 15–19 years (although this appears to be less of an issue in LAC than other areas) [20]. We examine disaggregated trends by age group (< 16 year, 16–17 years, and 18–19 years), wealth status, urban/rural residence to understand changes in basic characteristics of young first-time mothers. Wealth was measured using wealth quintiles. These are created using the DHS asset index, which is a measure of a household’s cumulative living standard based on ownership of selected assets, house construction and access to water and sanitation facilities. Our focus on first births reflects concerns over lack of progress, particularly as it is often masked by more optimistic trends in adolescent fertility rate. Confidence intervals at the 95% level were calculated for the percentage of first births by age group. We recognise the limitations imposed by the use of cross-sectional data: in particularly characteristics are recorded at time of survey not at time of birth, and this is discussed in more detail in the limitations section of the conclusion.

As discussed earlier, here is evidence that pregnancy outcomes for younger adolescents are worse than for older adolescents [4, 7, 8, 21, 22], so it was deemed important that the age groups were disaggregated in order to identify groups that were particularly vulnerable. Because the literature tends to use different age groupings it is difficult to conclusively identify the upper limit for the most vulnerable, but there is some evidence that disadvantage is particularly concentrated among mothers under the age of 16 years [4, 7, 8, 22], so this cut-off was chosen.

Inequalities are presented as ratios of poorest: richest quintile or rural: urban residence.

## Results

### Trends in adolescent motherhood

Table 2 shows the percentage of women aged 20–24 years at the time of the survey who reported a first birth before age 20 years (with confidence intervals in parentheses) in each age group for all three surveys for the five countries. Overall percentages for births to women under 20 years vary from 41% in the Dominican Republic to 27% in Haiti based on estimates from the most recent surveys. If we examine trends over time, we find that only Haiti has shown any evidence of a modest decline in first births to women under the age of 20 years across all three surveys (although confidence intervals still overlap for the 1994 and 2012 estimates, suggesting this could reflect sampling error). The estimates have remained static in Bolivia and Dominican Republic between the first and most recent survey and have increased in Colombia and Peru from 31 to 37% and 27 to 33% respectively. The Dominican Republic also has the highest percentage of births to women under 16 years at 7%, and again, only Haiti shows any evidence of a small reduction of births among the youngest (< 16 years) group from 4.6–3.2%. The Dominican Republic and Colombia have the highest proportion of all adolescent first births occurring in the < 16 years age group (16% for Colombia and 17% for Dominican Republic, based on the most recent survey), and in Colombia this proportion has actually increased over time. Due to the small changes over time, it is not really possible to make any firm comments about whether trends are markedly different for the periods from the baseline to the mid survey compared with between the mid and final survey, but there are no obvious generalizable patterns.

**Table 2:** Trends over time in % of women aged 20–24 years reporting first birth before age 20 years, disaggregated by age (CIs at the 95% in parenthesis)

Country	Year of first survey	Year of second survey	Year of third survey	Actual % change between baseline and final survey	Average % annual rate of change baseline to final survey year
Bolivia	1994	2003	2008		
< 16	5.2 (4.2–6.6)	4.5 (3.8–5.4)	5.2 (4.3–6.2)	0.0	0.0
16/17	13.3 (11.6–15.2)	14.5 (13.0–16.0)	14.9 (13.3–16.5)	1.6	0.9
18/19	19.2 (17.1–21.5)	21.8 (20.0–23.8)	17.3 (15.6–19.1)	−1.9	−0.7
Total < 20	37.7 (35.1–40.4)	40.8 (38.7–43.0)	37.3 (35.2–39.5)	− 0.4	− 0.1
Colombia	1990	2005	2010		
< 16	3.7 (2.6–5.2)	5.5 (4.8–6.2)	5.7 (5.1–6.4)	2.0	2.7
16/17	10.0 (8.2–12.1)	14.0 (12.9–15.2)	14.0 (13.0–15.0)	4.0	2.0
18/19	17.0 (14.3–20.1)	16.4 (15.2–17.7)	16.8 (15.8–17.9)	−0.2	−0.1
Total < 20	30.6 (27.4–34.0)	35.9 (34.2–37.6)	36.5 (35.1–37.8)	5.9	1.0
Dominican Republic	1996	2007	2013		
< 16	6.5 (5.3–8.0)	8.5 (7.4–9.7)	6.8 (5.2–8.7)	0.3	0.3
16/17	15.2 (13.2–17.3)	16.3 (14.8–18.0)	17.0 (14.7–19.5)	1.8	0.7
18/19	17.6 (15.5–19.9)	16.9 (15.3–18.6)	17.1 (14.9–19.7)	−0.5	−0.2
Total < 20	39.3 (36.5–42.1)	41.7 (39.5–43.9)	40.9 (37.7–44.0)	1.6	0.2
Haiti	1994	2005	2012		
< 16	4.6 (3.5–6.1)	4.3 (3.2–5.6)	3.2 (2.5–4.1)	−1.4	−1.7
16/17	10.4 (8.7–12.4)	10.8 (9.2–12.7)	9.7 (8.5–11.2)	−0.7	−0.4
18/19	16.5 (14.4–18.9)	15.1 (13.2–17.1)	14.4 (13.0–16.0)	−2.1	−0.7
Total < 20	31.6 (28.8–34.4)	30.1 (27.6–32.7)	27.3 (25.4–29.4)	−4.3	−0.8
Peru	1991	2007	2012		
< 16	3.3 (2.8–4.0)	3.5 (3.0–4.1)	3.5 (2.8–4.2)	0.2	0.3
16/17	9.1 (8.1–10.1)	10.3 (9.4–11.3)	11.1 (9.9–12.4)	2.0	1.2
18/19	14.5 (13.3–15.9)	14.9 (13.8–16.0)	17.1 (15.5–18.8)	2.6	0.9
Total < 20	26.9 (25.3–28.6)	28.7 (27.2–30.2)	31.6 (29.7–33.6)	4.7	0.9

Table 3 illustrates the percentage of women aged 20–24 years at time of survey reporting a first birth before age 20 years, disaggregated by age at first birth grouping and urban/rural residence for the baseline and final survey. If we examine the most recent surveys only, we find a higher proportion of adolescent first births occur in rural areas for all age groups in all countries. In some cases, these differences are quite marked: in Peru, women are twice as likely to give birth before 20 years in rural areas as in urban areas. In all five countries, the rural / urban differential is greater for the < 16 age group than for the 18–19 age group. This suggests that younger adolescent first births are particularly concentrated in rural areas. If we examine trends over time based on annual percentage rate of change for first births to women < 20 years, we see no consistent pattern. Percentages have risen in both urban and rural areas in Colombia. Conversely, percentages have risen in urban areas but fallen in rural areas in the Dominican Republic and Peru. However, in both these countries the rise in urban areas has been greater than the reduction in rural areas: in urban areas, adolescent first births rose at an annual percentage rate of change of 1.0 and 1.2 respectively for the Dominican Republic and Peru, whereas they only fell by − 0.7% and − 0.2%. Additionally, adolescent first births have risen in rural areas but fallen in urban areas in Bolivia, and have fallen for both areas in Haiti (from 28 to 22% in urban areas and from 35 to 32% in rural areas). In the Dominican Republic and Peru, the urban/rural differential has reduced for births to women < 20 years, whereas it has increased in Haiti, Bolivia and Colombia.

**Table 3:** % of women aged 20–24 years reporting a first birth before 20 years disaggregated by age and urban/rural residence: baseline and final survey

	Bolivia 1994				Bolivia 2008				% point change	Average annual % rate of change < 20
	< 16	16/17	18/19	< 20	< 16	16/17	18/19	< 20		< 20
Urban	4.3	10.7	19.0	34.0	3.9	11.4	14.4	29.7	−4.3	−0.9
Rural	7.1	18.5	19.5	45.2	8.2	23.5	24.3	55.9	10.7	1.7
Ratio rural/urban	1.7	1.7	1.0	1.3	2.1	2.1	1.7	1.9		
	Colombia 1990				Colombia 2010					Annual % rate of change
	< 16	16/17	18/19	< 20	< 16	16/17	18/19	< 20		< 20
Urban	3.0	8.5	15.3	26.8	4.5	12.8	15.1	32.4	5.6	1.0
Rural	5.8	14.3	22.1	42.2	10.7	19.0	23.9	53.5	11.3	1.3
Ratio rural/urban	1.9	1.7	1.4	1.6	2.4	1.5	1.6	1.7		
	Dominican Republic 1996				Dominican Republic 2013					Annual % rate of change
	< 16	16/17	18/19	< 20	< 16	16/17	18/19	< 20		< 20
Urban	5.0	12.7	15.2	32.9	5.5	16.6	16.8	39.0	6.1	1.0
Rural	9.7	20.3	22.7	52.7	10.7	18.1	18.0	46.9	−5.8	−0.6
Ratio rural/urban	1.9	1.6	1.5	1.6	1.9	1.1	1.1	1.2		
	Haiti 1994				Haiti 2012					Annual % rate of change
	< 16	16/17	18/19	< 20	< 16	16/17	18/19	< 20		< 20
Urban	4.9	10.2	12.5	27.6	2.5	7.6	12.2	22.4	−5.2	−1.0
Rural	4.4	10.6	20.4	35.3	3.9	11.7	16.5	32.1	−3.2	−0.5
Ratio rural/urban	0.9	1.0	1.6	1.3	1.6	1.5	1.4	1.4		
	Peru 1991				Peru 2012					Annual % rate of change
	< 16	16/17	18/19	< 20	< 16	16/17	18/19	< 20		< 20
Urban	2.3	6.1	11.4	19.8	2.4	8.4	14.8	25.6		1.2
Rural	7.1	20.1	26.1	53.4	6.8	19.7	24.4	50.9		−0.2
Ratio rural/urban	3.1	3.3	2.3	2.7	2.8	2.3	1.6	2.0		

Table 4 shows the percentage of first births to women aged 20–24 years at time of survey that occurred < 20 years, disaggregated by age at first birth and wealth quintile. There is a strong wealth gradient for all age groups in all five countries: women in the poorest quintile are at least twice as likely to report a first birth at age < 20 years as those in the richest quintile. Bolivia has the greatest differential for births under the age of 20 years: 66% of women in the poorest quintile have given birth by this age compared with only 12% of women in the richest quintile. The ratio between poorest and richest quintile tends to be smaller for the 18–19 age group and greater for the < 16 age group and can be extremely large in the youngest age group: in Colombia, the ratio of poorest to richest quintiles is 30 in Colombia and 13 in Bolivia. It is difficult to ascertain clear patterns over time by quintile, which may partly be due to limited sample size. However, in two countries, the ratio of richest to poorest quintile for births to women < 20 years has remained static, the ratio has increased in two countries (Bolivia and Haiti), and the ratio has decreased in one country (Peru).

**Table 4:** Trends in % of women aged 20–24 years reporting first birth < 20 years disaggregated by age group and wealth quintile

	Bolivia 1994				Bolivia 2008			
	< 16	16/17	18/19	< 20	< 16	16/17	18/19	< 20
Lowest	11.3	18.6	17.5	47.3	10.0	28.5	27.1	65.6
Second	6.2	14.6	26.7	47.4	9.3	19.5	25.1	54.0
Middle	5.5	18.0	21.7	45.2	6.2	18.1	20.6	44.9
Fourth	4.6	12.2	18.5	35.3	3.2	13.2	14.5	30.8
Highest	0.9	5.6	13.7	20.3	1.1	3.7	7.2	11.9
Ratio poorest/richest	12.6	3.3	1.3	2.3	9.1	7.7	3.8	5.5
	Colombia 1990				Colombia 2010			
	< 16	16/17	18/19	< 20	< 16	16/17	18/19	< 20
Lowest	11.8	16.7	24.4	52.9	13.7	22.0	23.5	59.1
Second	2.3	15.8	21.8	39.9	7.1	18.2	22.5	47.8
Middle	4.9	8.8	18.0	31.7	5.0	14.4	16.5	35.9
Fourth	1.7	8.4	13.3	23.4	3.8	9.7	14.8	28.2
Highest	0.4	3.4	10.9	14.6	0.8	7.5	8.2	16.4
poorest/richest	29.5	4.9	2.2	3.6	17.1	2.9	2.9	3.6
	Dominican Republic 1996				Dominican Republic 2013			
	< 16	16/17	18/19	< 20	< 16	16/17	18/19	< 20
Lowest	11.6	31.4	25.8	68.8	10.6	28.9	23.6	63.0
Second	9.9	17.5	24.7	52.2	10.3	21.9	20.7	52.9
Middle	7.7	16.9	17.0	41.6	6.3	16.9	16.9	40.1
Fourth	2.9	9.6	17.4	29.8	4.5	10.9	13.5	28.9
Highest	3.0	6.6	8.4	18.1	2.4	7.4	11.7	21.4
poorest/richest	3.9	4.8	3.1	3.8	4.4	3.9	2.0	2.9
	Haiti 1994–95				Haiti 2012			
	< 16	16/17	18/19	< 20	< 16	16/17	18/19	< 20
Lowest	7.6	12.8	21.4	41.8	4.2	13.6	19	36.8
Second	5.7	12.8	24.8	43.3	4.1	10.2	22.2	36.5
Middle	4.5	10.7	18.5	33.7	3.5	12.8	17.2	33.5
Fourth	5.8	10.6	16.5	32.8	4.0	10.6	12.3	26.8
Highest	1.7	7.6	8.7	18.1	1.3	4.4	7.4	13.0
poorest/richest	4.5	1.7	2.5	2.3	3.2	3.1	2.6	2.8
	Peru 1991–92				Peru 2012			
	< 16	16/17	18/19	< 20	< 16	16/17	18/19	< 20
Lowest	7.6	24.1	28.5	60.2	7.7	23.3	25.2	56.2
Second	5.8	13.6	23.1	42.6	6.4	18.5	25.0	50.0
Middle	3.8	8.9	16.2	29.0	2.3	9.4	16.2	27.9
Fourth	0.7	4.4	9.4	14.5	1.7	6.9	15.2	23.8
Highest	1.3	1.9	4.5	7.7	1.4	3.1	8.0	12.5
poorest/richest	5.8	12.7	6.3	7.8	5.5	7.5	3.2	4.5

## Discussion

Our study set out to answer two questions:

1. What is the age distribution of first births to adolescents, and have the percentages of younger and older adolescent girls (10–14 and 15–19 years respectively) changed since 1990?
2. How do the socio-economic characteristics (wealth and urban / rural residence) of young women having a first adolescent birth differ between younger and older adolescents and how have these characteristics changed over time?

Regarding the first question, our study finds very little evidence of marked progress in reducing adolescent first births in any of the countries. Indeed, in two countries (Colombia and Peru) there has been an increase and in a further two countries (Dominican Republic and Bolivia) the percentage has stagnated. It is particularly concerning to note the persistence, and in the case of Colombia increase, in births to young women under the age of 16 years. The implications of this are underlined by evidence that the risks of ill health and mortality to the mother and child associated with adolescent births are most concentrated within this age-group [4, 8].

Regarding the second question, our study clearly shows that adolescent first births remain concentrated among poorer and rural women, and in some countries these differentials have increased over time. Inequities between both wealth and place of residence have only decreased in Peru (although because wealth is measured at the time of survey, it is impossible to state categorically whether poverty is a cause or a result of adolescent motherhood). Younger adolescent mothers are also more likely to be poor and live rurally when compared to their older counterparts [4, 8], which further adds to their vulnerability [23].

Our finding of the limited reduction in adolescent first births in the five countries studied are consistent with those from other studies in the LAC regions, which describe stagnation or increases in the levels of teenage motherhood. For instance, a study by Rodriguez-Vignoli [17] found stagnation or increases in the proportion of women aged 15–19 years who were pregnant or had had a first child in a number of countries within the region during a similar period. It is interesting to note that stagnation in Dominican Republic and Bolivia occurs within the context of overall decline in ASFR 15–19 years . This gives further support to Rodriguez-Vignoli’s argument that such declines in LAC are driven by a reduction in the number of births to each adolescent, not the proportion of young women who become adolescent mothers in the first place [13]. Batyra’s study in Colombia [24] also demonstrates how successive cohorts of women are increasing the timing of subsequent births.

This lack of progress is clearly concerning: not only does this negatively impact the health and wellbeing of young women and their children [4, 7], it will have wider repercussions for families and communities and will also hinder progress towards the Sustainable Development Goals (SDGs). The adolescent fertility rate is an indicator for the third SDG: Ensure healthy lives and promote well-being at all ages. However, reducing adolescent pregnancies will also potentially contribute more widely to the SDGs through reducing the cycle of deprivation, as well as positively impacting on the goals focussing on poverty, hunger, quality education, gender equality and decent work and economic growth [5, 25–28].

The concentration of adolescent births to poorer mothers identified in this study is also in line with other work from the region [29, 30] that finds socio-economic inequalities persist in adolescent pregnancies. Bozon et al. [29] reflects that the traditional feature of marked inequality within Latin American societies translates into gross inequities in sexual experiences and outcomes. Further, our finding that younger adolescent mothers are also more likely to be poor and rural residents compared to their older counterparts are in line with similar findings in the East African context [23].

Our study clearly indicates the need for increased efforts to address adolescent births within these countries. As adolescent pregnancy is often underpinned by social, economic and cultural factors, it is increasingly understood that individual-level focused interventions are likely to have limited impact, and that it is vital to involve families, communities, and wider society [31]. As Caffe et al. have noted, addressing the issue of adolescent first births in LAC is likely to require a broad and multisectoral approach, and strategies that address both the proximal and the distal determinants of adolescent fertility within the LAC context have been widely supported [32].

Education is seen as a key component of multisectoral approaches to reducing adolescent births, and attempts to increase school enrolment through conditional cash transfers have had some success in both Colombia and Peru: both have improved school enrolment and there is some indication of reduced adolescent pregnancy [14]. However, there are no simple solutions. The relationship between education and adolescent pregnancy in LAC is complex, and advances in education coverage in some LAC countries have failed to reduce teenage pregnancies. Rogriguez-Vignoli and Cavenaghi’s study [33] found that the impact of education on adolescent pregnancy was not linear, and the threshold for impact increased from 5 years in the 1990s to 7 years in more recent periods. They highlight problems in the educational system whereby inequalities in quality persist, and where educational thresholds for employment have increased as much as, or more than, increases in average education, meaning many more disadvantaged young people are still excluded from the job market. In addition, complex social and cultural factors mitigate the relationship between education and adolescent pregnancy in LAC, including gender relationships and low aspirations.

In addition, adolescents need sexuality education that provides information and skills to protect themselves and avoid adverse health and social outcomes. The social and cultural aspects of adolescent pregnancy underline the importance of a holistic approach to sexuality education that addresses social and cultural norms including gender inequality and relationship dynamics [31]. Within the five countries there is wide variation in approaches to adolescent sexuality education: while Colombia has adopted many of the principles of comprehensive sexuality education (CSE), Peru and the Dominican Republic continue to focus on a much narrower curriculum [34].

Thirdly, adolescents need to be able to obtain contraceptive information and services, including emergency contraception. Rodriguez-Vignoli [13] highlights that lower rates of adolescent pregnancies in other countries with high rates of adolescent sexuality are driven by increased access to contraception and abortion. The First Latin American and Caribbean Regional Conference on Population and Development, held in Montevideo in August 2013 provided a forward-looking consensus, which agreed to the implementation of:

*“…comprehensive, timely, good-quality sexual health and reproductive health programmes for adolescents and young people, including youth-friendly sexual health and reproductive health services with a gender, human rights, intergenerational and intercultural perspective, which guarantee access to safe and effective modern contraceptive methods, respecting the principles of confidentiality and privacy, to enable adolescents and young people to exercise their sexual rights and reproductive rights, to have a responsible, pleasurable and healthy sex life, avoid early and unwanted pregnancies, the transmission of HIV and other sexually transmitted infections, and to take free, informed and responsible decisions regarding their sexual and reproductive life and the exercise of their sexual orientation”*) [35]*.*

However, despite promising moves towards improving sexual and reproductive rights and services in the region, barriers remain for marginalized populations, including adolescents. Socio-economic inequalities in access remain problematic [36], and our findings relating to persisting socio-economic differences highlight the importance of improving access for the poorest and rural residents.

Finally, gender based violence (GBV) needs to be addressed effectively. GBV is a significant problem in much of LAC, and many of the adolescent births may result from coerced sexual contact or abuse from an adult male [28]. It is estimated that between 18% (Dominican Republic) and 43% (Bolivia) of women aged 15–19 years have experienced intimate partner violence (IPV) in the five countries within our study [37]. In all five countries, adolescent respondents aged 15–19 years had an increased risk of experiencing IPV in the previous 12 months than women aged 30–39 years [37]. Additionally, in some countries, reports of forced sexual debut are common. The link between IPV and adolescent pregnancy is clearly recognised in a number of strategies within the region, including the Mexican Adolescent Pregnancy Strategy [38], and most countries in the region now have national commissions to address violence against women. Some countries face particular challenges: in Haiti, where 21% of women aged 15–24 described their first experience of intercourse as “forced” or “rape” [37], a number of reports and studies point to increased SGBV, transactional sex and adolescent pregnancies among displaced communities and populations most affected by the earthquake in 2010 [39, 40]. Additionally, in Colombia many women are still suffering the effects of conflict and displacement, which creates specific problems and hinders progress [41].

Latin American countries have made strides in strengthening legislation around this issue, and there have been promising initiatives to promote change among law enforcement agencies, as well as developing interventions to support victims. A number of community- and school-based programmes have demonstrated success in changing violence-related attitudes and behaviours, and the gender norms that underpin this problem [42], which must be viewed as a vital component of strategies to reduce adolescent births.

Our study has a number of limitations, which reflect constraints in the data on which the analysis is based. Our study only relates to live births, and does not cover spontaneous or induced abortion. An estimated four million induced abortions take place in Latin America each year, despite the fact that it is highly restricted in all countries [43]. The vast majority of these abortions are likely to be unsafe. There is limited understanding on adolescents’ use of abortion in this region, and while data are collected in DHS (albeit without distinguishing induced abortion from other pregnancy loss) it is of very poor quality [44] so has not been included in this study.

As mentioned in the methodology section, there are a number of limitations associated with cross-sectional data that influence the interpretation of the findings. In particular, the characteristics of place of residence and wealth are measured at the time of the survey rather than at the time of the birth. This creates problems in interpretation: poverty could be either a driver or a result of adolescent motherhood. Furthermore, young women may migrate following a birth (either to live in a different household or to find employment) which also means the analysis of urban / rural residence should be interpreted with some caution.

It must also be noted that our data is somewhat dated, and may not reflect recent improvements resulting from efforts to address this issue or from wider social and economic changes. The adolescent births identified in the 20–24 year age group could have occurred up to about 10 years before the date of the survey, and some of the surveys themselves are already somewhat dated. At the time of writing the data had not been released for the Colombia 2015 DHS, but the preliminary report suggests that the proportions of women aged 15–19 at the time of survey who have either begun childbearing or are pregnant have fallen [45]. It is vital that new survey data is comprehensively analysed as it is published and additional sources are explored to ensure a contemporaneous picture of ongoing trends in the region.

## Conclusion

Adolescent motherhood continues to be an issue for concern in the countries we studied, with little or no progress in reducing the percentage of women who give birth before the age of 20 years. This not only puts the health of many women and their children at risk and limits their educational and economic opportunities, but also impacts more broadly on families, communities, and national development as a whole. Adolescent births continue to be concentrated amongst the poor and those living in rural areas. Additionally, there is a concerning persistence in births to very young adolescents under 16 years of age, who are likely to experience the most severe risks and disadvantages. Efforts to provide sexual health information and services for young people need to accelerated, along with strategies to improve education and employment opportunities and further strengthen legal frameworks to protect those at risk of abuse and exploitation. Additionally, greater efforts need to be made to disaggregate adolescent pregnancy and sexual health data in order to measure progress in vulnerable groups and ensure that the SDG intention that no one is left behind becomes a reality.

## Abbreviations

DHS: Demographic and health survey
LAC: Latin America and the Caribbean
PLANEA: Andean plan to prevent teen pregnancy

## Footnotes

1 We define adolescent as between 10 and 19 years old
